# Determinants of Nurturing Care Behaviours in Malawi: An Ethnographic Study

**DOI:** 10.1111/mcn.70076

**Published:** 2025-07-27

**Authors:** Roschnik Natalie, Jupp Dee, Stein Danielle, Abisaputra Iqbal, Adhima Rizqan, Chasowa Sarah Kudeko, Ellis Steven, Gunda Rita, Indra Yeni, Kadawati Prisca, Kandulu Blessings, Koirala Neha, Kulemba Anthony, Lundu Chisoni, Lupafya Phindile, Mnyawa Judith, Mshanga Steven, Nowa Mphatso, Nkhonjera Jean, Phiri Brenda, Sambani Sungeni, Tobing Deborah, Zintambira Fatsani, Keiser Olivia, Gladstone Melissa

**Affiliations:** ^1^ Save the Children UK London UK; ^2^ Empatika Jakarta Indonesia; ^3^ Save the Children International Lilongwe Malawi; ^4^ Ntcheu District Council Ntcheu Malawi; ^5^ Balaka District Council Balaka Malawi; ^6^ Institute of Global Health University of Geneva Geneva Switzerland; ^7^ University of Liverpool Liverpool UK

**Keywords:** child, child development, diet, food insecurity, gender equity, hygiene, Malawi, malnutrition, mental health, poverty, pregnant women

## Abstract

In Malawi, 38% of children under five are stunted, and only 36% are developmentally on track. An ethnographic study using the Reality Check Approach was conducted in four villages in Ntcheu and Balaka districts in Malawi to understand caregiving‐related drivers of malnutrition and poor child development. Researchers immersed themselves in 12 rural households with a pregnant woman or a child under 2 years for 4 days and nights, and gathered information through informal conversations, participation in daily activities, observations, and group sessions. They assessed current behaviours and the factors driving these behaviours in five recommended nurturing care domains essential for a child to both survive and thrive. The study revealed that children were not receiving the nurturing care they needed. Caregiving‐related issues identified included poor maternal, child, and family diets; inadequate infant and young child feeding; poor hygiene environment and practices; low use of health and nutrition services; limited responsive care, stimulation and learning opportunities, and early pregnancies. Caregiving determinants included chronic poverty and food insecurity, climate and economic shocks, low access to quality health, nutrition and early childhood development services, poor maternal wellbeing, gender inequality and harmful social norms. The research underscores the need for a multi‐sector approach to improve maternal and child nutrition and development. It also stresses the importance of understanding the contextual determinants of caregiving behaviours to inform multi‐sector programmes and policies and meet Malawi's Sustainable Development Goals and World Health Assembly targets for maternal and child health, nutrition, and development.

## Summary

- Young children in Ntcheu and Balaka districts are not receiving the nurturing care they require to grow and develop to their full potential.
- Key caregiving‐related challenges include poor maternal, child, and family diets; inadequate infant and young child feeding; poor hygiene environment and practices; low use of health and nutrition services; limited responsive care, stimulation and learning opportunities, and early pregnancies.
- These caregiving practices are influenced by chronic poverty and food insecurity, climate and economic shocks, low access to quality health, nutrition and early childhood development services, poor maternal wellbeing, gender inequality and harmful social norms.
- Multi‐sectoral programmes that address the contextual drivers of caregiving practices are needed to prevent malnutrition and improve child development in Malawi.

## 1 Introduction

The adoption of the Sustainable Development Goals has brought global recognition that future health, prosperity, peace, and freedom of children goes beyond children surviving to children thriving (United Nations 2015). The Nurturing Care Framework identifies five domains that children need to survive and thrive. These include safe and nutritious food; health care; responsive care; learning opportunities, safety and security (Black et al. 2020; World Health Organization 2018). While these domains are internationally recognised and supported by global evidence (Prado et al. 2019), the factors that influence these domains vary widely by context. Understanding these contextual drivers is crucial to design multi‐sector programmes that are tailored to each context and target the most relevant caregiving practices and behavioural drivers to effectively prevent malnutrition and improve child growth and development.

In Malawi, the prevalence of stunting is high at 38% (National Statistical Office Malawi and ICF 2024), above sub‐Saharan Africa's 29% average (United Nations Children's Fund UNICEF 2020). 63% of children under 5 years are anaemic, and only 36% of children aged 3–5 years are developmentally on track in at least three out of four Early Childhood Development Index domains (National Statistical Office NSO 2021). Children with malnutrition in Malawi are more likely to have lower development scores compared to well‐nourished peers (Gladstone et al. 2010). Quantitative studies have identified a wide range of factors associated with stunting in Malawi, which include maternal factors (age, education, income, health, diets) (NSO, 2017); gender inequality (Rico et al. 2019; Chirwa and Ngalawa 2009); household food insecurity (Kang et al. 2019); child health and disease (chronic infections, recurrent malaria and diarrhoea) (Maleta et al. 2021; Scarcella et al. 2016; V. Doctor and Nkhana‐Salimu 2017), and inadequate caregiving practices (hygiene, infant and young child feeding and low use of services) (Osendarp et al. 2019; Titilayo et al. 2016; NSO 2017; National Statistical Office NSO 2020; National Statistical Office NSO 2021). Child development of children under 2 years was associated with caregiver diet and empowerment (Bliznashka et al. 2024). While these quantitative studies are useful in identifying potential drivers of malnutrition, they do not unpack how these factors influence caregiving practices. Some qualitative studies have been conducted to understand caregiving behaviours in Malawi, but they have either been limited to descriptions of existing knowledge and practices (Mseu et al. 2014) or have focused on just one driver, e.g. gender roles in child nutrition (Mkandawire et al. 2022). Only one other study has attempted to understand the barriers and opportunities for improving nurturing care behaviours (Gladstone et al. 2018), but the focus was mainly on early child development‐related domains. All the studies used more traditional qualitative research methodologies (Focus Group Discussions, in‐depth interviews), and none used experiential and observational methods to assess drivers of caregiving behaviours from the caregiver perspective.

The goal of this study was to understand the complex drivers of caregiving behaviours across the five nurturing care domains in Ntcheu and Balaka districts of Malawi, using participant and naturalist observation and engagement methods to help fill a gap in contextual evidence around caregiving drivers of malnutrition and poor child development, which in turn can be used to inform multi‐sectoral stunting prevention and child development programmes in Malawi and similar contexts.

## 2 Methods

This study was conducted as part of the MAZIKO research project (Save the Children 2023) and followed a research protocol developed by Empatika (Empatika 2025) in collaboration with Save the Children (Empatika 2022).

### 2.1 Theoretical Framework and Research Method

The study used the Reality Check Approach (Burns et al. 2021; Shah 2018), an “ethnographic immersion” exploratory research method, which includes an immersion in the community to observe and participate in daily activities. This method was selected because it provides rich, detailed data that captures subtleties often missed by traditional qualitative research methods. It also prioritises participants' perspectives and considers all influencing factors, making it ideal for understanding complex social phenomena.

### 2.2 Study Location

The study was conducted in Ntcheu and Balaka districts in Malawi, where stunting rates are 36% and 45% respectively (National Statistical Office NSO 2021). Four communities were selected, two in each district, based on: geographical diversity (to represent different districts, income and livelihood zones); presence of a Community Based Childcare Centre (CBCC); and distance to markets and services (close and far). All selected communities were poor, rural and reliant on subsistence agriculture, with Balaka being hotter and drier than Ntcheu. The study was conducted between February and April 2022, a period characterised as the lean season when the least food is available.

### 2.3 Researcher Training and Characteristics

The research was led by a team from Empatika (Empatika 2025), an organisation specialising in people‐centred ethnographic research, who trained and mentored local researchers. The researchers were selected from a total of 26 Malawian national and district‐based NGO and government technical staff, who were trained over a week on qualitative and participatory research skills, and attitudes and behaviours needed for conducting respectful and productive immersions. The training included guidance and practical exercises on informality, power, bias, positionality, rigour, triangulation, and how to have authentic conversations, deepen insights with observations, and connect with people through shared experiences. From the 26 trained, 12 researchers were selected by the Empatika team based on training performance, and their ability to show curiosity, openness, connect informally, and set aside technical and cultural biases. Supporting Information S1: 1 summarises the researcher characteristics. All 12 researchers completed a child protection and safeguarding training and signed a form committing to avoid all sexual, exploitative, physically or mentally abusive or humiliating relationships or acts, and ensure children, and communities are protected from harm and treated with respect.

### 2.4 Sampling and Community Entry

The researchers entered the communities without prior contact to minimise community bias and pre‐selection of households. On arrival, researchers met the Village Leader to explain the research purpose and get their approval to conduct the study. Researchers engaged with households through informal conversations to identify those suitable for participation in the study. Suitability was determined in situ based on the following criteria: presence of a pregnant woman or a child under 2 years, preference for multi‐generational households or extended family, and families in socio‐economically disadvantaged or marginalised groups. Supporting Information S1: 2 provides details on the number of households approached, the number unwilling to participate and the reasons given. All 12 households who agreed to participate remained in the study for the full 4 days and were given food and soap as compensation.^1^

### 2.5 Data Collection

Researchers lived in each household for 4 days and nights, gathering information through informal conversation, observation, and participation in daily activities or focused exercises with household members, neighbours, community members, or frontline workers met over the 4‐day period (see Table 1). A one‐page memory aid with six areas of conversation was used by researchers to guide their discussions and activities and ensure consistency of topics covered across researchers (See Supporting Information S1: 3). These areas of conversations were developed in consultation with Malawi district government and Save the Children field and technical staff, drawing on existing evidence, experience and government maternal and child health, nutrition and development behavioural priorities. No notes were taken in front of household and community members to keep discussions informal. Instead, researchers recorded notes, numbers, or quotes in field diaries when they were alone.

**Table 1:** RCA methods used.

Tool	Method	Information gathered
informal conversation	Informal, spontaneous conversations used iteratively throughout the day and while participating in daily activities and chores, to optimise opportunities for triangulation.	Behaviours and behavioural drivers for key “areas of conversation” (Supporting Information S1: 3)
Family tree	Co‐creating a family tree, noting age, gender, who lives away/in the house, relationships and childcare responsibilities.	Understand who takes responsibility for different needs of babies and childcare.
Ranking	A pairwise ranking where household members select and compare typical foods based on perceived health and nutritive value, cost, consumption frequency, liking or disliking, typical and special meals, and seasonal availability.	Understand food preferences and eating habits, including seasonal availability, consumption frequency and reasons for prioritising certain foods.
Food diary	Researcher completes a full day's food diary of pregnant, breastfeeding mothers or children under 2 years, through observation.	Assess what is actually eaten, how food is prepared and the eating/feeding behaviours and environment.
Seasonality diagram	Mapping of available foods throughout the year and discussing coping strategies with household members.	Understand coping mechanisms and alternative strategies to managing seasonal challenges.
Observation and experience	Researchers observe and experience the realities of living in these communities by accompanying people to services, meetings, markets, field, etc.	Gather additional insights that do not come up in discussions.

### 2.6 Data Upload and Analysis

The data upload and analysis were undertaken by an Empatika Research Lead, Technical Advisor and People‐Driven Development Lead. Immediately after the immersion phase, they held three 2‐day debrief and documentation sessions, one for each location with the three researchers from that location to “download” all the information gathered over the 4‐day period. The debrief sessions were structured by areas of conversations (with themes and sub‐themes) and built upon the photos, notes and activity outputs (seasonal calendars, food dairies, family tree, ranking exercises) that the researchers had gathered. During the sessions, findings were triangulated to confirm issues and explore differences between the researchers' accounts. The Empatika Research Lead and Technical Advisor then organised the data into written and coded debriefing notes, photographs, annotated visuals, case stories, field diaries and socioeconomic profiles for households and communities to form a comprehensive “data set.” The analysis process followed a “grounded theory” and Framework Analysis Approach (Ritchie et al. 2003), which begins with a familiarisation of the de‐briefing notes, case studies and household and community descriptions. Key issues, themes and categories were then identified and hypotheses and theories constructed through inductive reasoning to draw inferences from the charted summaries. The Lead Research and Technical Advisor worked both independently and jointly to identify and test emerging themes and findings, to add credibility and rigour to the findings.

### 2.7 Community Feedback and Validation

A People Driven Design phase (Empatika 2020) was conducted 2–3 weeks after the immersion phase, to share back and validate findings from the research with the community and explore solutions to the main issues identified. Empatika trained eight of the 12 immersion phase researchers to lead 3‐h workshops over 3 days with community members. The workshops focused on four key issues: (1) limited stimulation and learning opportunities; (2) poor hygiene environment in the household; (3) low diet diversity; and (4) early introduction of foods and fluids. The researchers were assisted by NGO field staff who helped facilitate and take notes during the workshops. Participants included immersion study households and other relevant community members, e.g. breastfeeding mothers to discuss breastfeeding, elder siblings and grandmothers to discuss stimulation and learning opportunities. Findings from the immersion research were shared with the participants, who were asked to reflect, agree, disagree, or add. Solutions were then identified through collaborative activities. Key reflections and quotes from these workshops were transcribed and have also been included in the results below. The solutions identified were used to create a set of small doable behaviours to promote through the MAZIKO programme, which are reported elsewhere (Empatika 2022).

### 2.8 Ethics Statement

Ethical approval was obtained as part of a larger trial approval: from the institutional review boards of the University of Malawi (protocol number: P. 02/22/128), the International Food Policy Research Institute (protocol number: 7490), and by PHND – 22 – 0214 on 27th February 2022. Researchers explained to selected households that participation was voluntary, that their name would remain confidential, and that they could communicate any concerns about sharing information. Household members were then asked for verbal consent to participate in the study. Approvals were also received from the Village leader.

## 3 Results

### 3.1 Characteristics of Sample

Table 2 describes the characteristics of each village and sampled household involved in the immersion phase and participants in the validation workshops. Across the 12 households, researchers interacted with 13 mothers (including two 18 y old mothers), five fathers, four grandmothers, 15 school age children (5–15 y), four preschool children (3–5 y), six children aged 6 months to 2 years and seven babies under 6 months and two pregnant women. Researchers also interacted with another 378 community members (283 women and 95 men)—neighbours, extended family, traditional and religious leaders, medicine sellers, food vendors, Savings and Loan Group members, health volunteers, former traditional birth attendants, and CBCC volunteers. During the community feedback and validation workshop, they interacted with 26 mothers, 10 grandmothers, two teenagers and one father.

**Table 2:** Characteristics of the village, households and community members engaged with.

Location	Village characteristics	4‐day Immersion research	Community feedback/validation workshop
Balaka West TA Chanthunya	∘ ~460 households ∘ Majority Yao ethnicity and Muslim religion ∘ 9 km from main road ∘ Prone to floods ∘ 1.5 h walk to market ∘ 1.5 h walk to health clinic	**Household 1** Grandma, mother (30 y), 3 children (14 y, 6 y, 15 months). Husband works away **Household 2** Grandma (83 y), mother (23 y), 1 child (5 y) and 1 baby (6 weeks) **Household 3** Grandma (50 y), pregnant daughter (18 y), husband works away	**Topic: Stimulation and learning** 3 mothers (23–30 y) 2 grandmas (55,56 y) 2 teenagers (14 y girl, 17 y boy) 1 father
Balaka East TA Amidu	∘ 1645 households ∘ Majority Christian, Ngoni and Chewa ethnicity ∘ 2 h walk from main road ∘ Prone to floods ∘ 4‐5 km from market ∘ 4‐5 km from clinic (including river crossing)	**Household 1** Mother (23 y), 1 child (2 y) and baby (5 m), Father migrated to South Africa **Household 2** Grandma (78 y) Mother (37 y) 4 children (14 y, 12 y, 8 y, 6 y) and baby (3 weeks) Husband in South Africa **Household 3** Mother (29 y), 2 children (12 y, 17 months) 17 y son in South Africa, Father sells headphones 9 km away;	**Topic: Poor hygiene in the home** 6 mothers (18–37 y) 6 grandmas (65–79 y)
Ntcheu N.TA Masasa lower	∘ 3 households ∘ Majority Ngoni ethnicity and Christian religion ∘ 45 min walk from main road ∘ 45 min walk from market ∘ 45 min walk from clinic	**Household** 1 Mother (33 y), father (37 y), 5 Children (13 y, 11 y, 9 y, 3 y, 2 y) **Household 2** Pregnant woman (25 y), husband (27 y) **Household 3** Mother (24 y), Father (30 y) 2 children (6 y, 3 y), 1 baby (2 weeks)	**Topic: low diet diversity** 8 mothers (20–30 y) 2 grandmas (50–60 y).
Ntcheu SW TA Mpando	∘ 3 households ∘ Majority Ngoni ethnicity and Christian religion ∘ 15 km from main road ∘ 12‐15 km from market ∘ 12‐15 km from Clinic (3‐4 h walk)	**Household 1** Mother (25 y), father (62 y) 7 children (18–41 years), 18 y daughter had 2 children (3 y, 18 m) **Household 2** Divorced mother (42 y) 2 children (13 y, 9 y) and 2 × 1 m old twins) **Household 3** Father (51 y) Mother (30 y), Breastfeeding mum (22 y), 3‐day‐old baby	**Topic: early introduction of foods and fluids** 9 breastfeeding mothers (18–42 y)

### 3.2 Findings by Nurturing Care Domain

**Figure 1:**
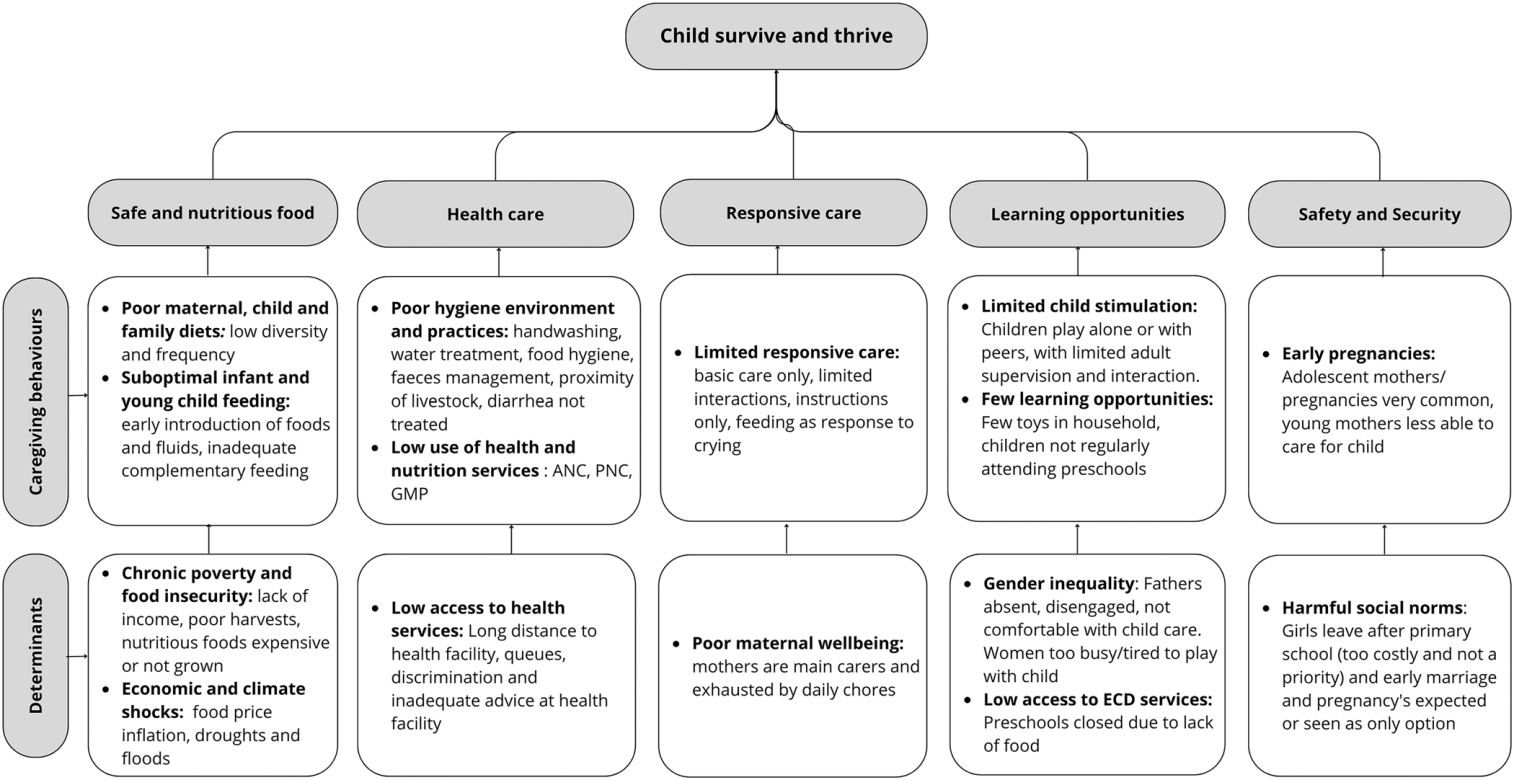
Summary of drivers across 5 nurturing care domains.

Figure 1 summarises the key findings from the immersion and community feedback/validation phases by nurturing care domain and detailed findings are summarised below.

### 3.3 Safe and Nutritious Foods

#### 3.3.1 “Here We Don't Have Options in Terms of What to Eat”

Researchers observed that in all 12 study households, families only ate two meals a day (in the afternoon and evening), which usually consisted of nsima (a thick/hard porridge made of maize flour and water) with boiled leaves (mustard, pumpkin, moringa or amaranthus). Mothers reported that they only ate two meals a day because they did not have sufficient maize or cash to have breakfast as well and were *“too busy”* to prepare supplementary meals. The researchers observed that the leaves were often brown and limp due to overcooking. Mothers reported adding oil, tomato, small fish, groundnut powder, soya chunks or legumes to the meal, when available and if the families could afford it, which was rare. Researchers observed mice, crickets and other insects being eaten in some locations. Only a few children in Ntcheu were observed having snacks and these were either roasted maize or banana fritters (banana fried with maize flour, sugar and salt). Livestock (goats, chickens, ducks) were described by families as being *“too valuable to eat”* and were instead used as savings or assets to sell when cash was urgently needed.

Here we don't have options in terms of what to eat.

(Family member, People Driven Design session, Balaka West)

We can only eat what we have.

(Mother, Ntcheu North)

Household members said that the family diet was influenced by the crops they grew and what could be found, traded or purchased locally which varied by season. Table 3 summarises six seasonal calendars developed by households (two in Balaka and four in Ntcheu). It shows that December to March is the most difficult time. As this study took place in February, many families said this was a particularly challenging time when food was *“never enough”*, and they “*struggled to find enough food.”*

**Table 3:** Seasonal availability of food.

		Seasons		
		**Season of plenty** (Apr‐Aug) When staple crops are harvested, excess crops are sold for cash, and when fruit, beans and peas are available	**Getting harder** (Sept‐Nov) When families start using their food stores and less food is available	**Lean season** (Dec‐March) When the least food is available, and the least food is consumed. Stocks of maize are exhausted and the little cash available is used to buy fertiliser and other agricultural inputs for the upcoming planting season.
Foods availability	Staple			
	Legumes			
	Veg			
	Fruit			
	Oil			
	Animal			

*Note:* light grey: food is available; medium grey: food is less available; dark grey food is not available.

Families reported declining crop production due to worsening soil fertility, erratic weather, and a declining water table, as the main reasons for the lack of food. In Ntcheu North, families said the rains were becoming unpredictable, affecting planting timing, shortening the growing season, and forcing families to plant maize 2 weeks later than usual, delaying harvest.

#### 3.3.2 “When We Eat, Our Breasts Are Plump, Full of Milk, Otherwise They Seem Withered”

Researchers observed that pregnant and breastfeeding mothers ate the same meals as the family ‐ two meals of nsima a day, with the first meal around 1 pm, after a morning working in the field or doing chores. One breastfeeding mother in Balaka West ate only one meal of nsima daily.

We get what we can from the field and eat the same food as the rest of the family.

Mother, Ntcheu West

A pregnant mother in Balaka West worried about not eating enough food while pregnant and her baby not growing. The hardest months to breastfeed were the lean months (Jan‐March), because *“we are hungry ourselves.”*

When we eat, our breasts are plump, full of milk, otherwise they seem withered.

Breastfeeding mothers, People Driven Design, Ntcheu West

The most common food taboo mentioned for pregnant women was eggs, considered *“unacceptable”* and potentially causing baldness in babies. Other beliefs, village or family‐specific, included avoiding tomato or pepper (Ntcheu North) and eating okra for easy delivery (Balaka West).

#### 3.3.3 “It Is Hard to Breastfeed When You Yourself Are Hungry”

Mothers claimed they exclusively breastfed their child for the first 6 months. However, one researcher in a household with a 2‐month‐old observed the mother giving her baby 4–5 spoons of sugar dissolved in water. When asked why they gave young babies food and liquids, mothers said it was because their babies were hungry, and their breastmilk wasn't enough.

It is hard to breastfeed when you yourself haven't eaten enough.

Mother, Ntcheu West

Mothers said advice from health workers to eat well while breastfeeding made them more concerned that their milk was insufficient and concluded their babies cried because they were hungry.

A playing baby is a happy baby, but a crying baby is hungry.

Breastfeeding mothers, People Driven Design, Ntcheu West

Those mothers who had started giving their babies food, they were observed giving them porridge of maize flour and water, which they said they thickened as the child got older by adding groundnut powder, tomato, or soya when money was available. No mothers were observed adding leaves or fruit. Since sugar was too expensive, babies' porridge was flavoured with salt, which researchers noticed babies didn't seem to like and often didn't finish.

### 3.4 Health Care

#### 3.4.1 “PNC Clinics Are a Waste of Time”

- *Health services are too far*.
  Antenatal Clinic (ANC) and Post Natal Care (PNC), which includes Growth Monitoring and Promotion (GMP), were the main maternal and child health services that mothers mentioned accessing, but did not attend monthly as recommended. In Balaka, PNC was available through village outreach clinics, but in Ntcheu, both ANC and PNC were only at hospitals or health facilities, which were too far. No mother mentioned receiving a home visit by a health worker or volunteer. Care Groups (volunteer mother support groups) had operated in all four study locations, but only one in Ntcheu North was still active.
  In Ntcheu West, mothers said they had to walk 3‐4 h each way to attend ANC or PNC. In Ntcheu North, they had to wade through a river, which was challenging when pregnant or carrying a small baby. Some said they would get a lift on a motorbike, costing MWK 1000 (USD1.2)^2^ each way. All women gave birth at the clinic or hospital (to avoid an MWK 15,000 ‐USD18) fine), but this meant (in Ntcheu West) staying near the hospital 3 weeks before their due date, to avoid travelling when in labour.
- *Long waiting times and inappropriate advice*.
  Women said that once their child was fully vaccinated, PNC sessions had little value, and the waiting times were too long just to get their babies weighed: *“PNC clinics are a waste of time,”* said a mother in Balaka East.
  Some mothers received inappropriate advice. One mother for example said a nurse suggested feeding her newborn spoons of warm water to help them stay warm. Other mothers said the advice was not understandable or feasible:

I was advised to eat the flesh of the cassava fruit, starchy food and water that rice has been soaking in to generate breast milk. But I was not able to follow the advice on rice, since it didn't grow nearby, and I could not afford to buy it (500–700 MKw/Kg).

Mother, Balaka East

Some mothers said that the advice to eat six food groups to produce enough milk for their babies made them more worried that their milk was not enough (since the advice was not feasible), and then gave their babies other foods, instead of exclusively breastfeeding.

- *Fines and discrimination*.
  The Malawi government recommends husbands accompany pregnant wives to ANC, which women said was challenging as husbands are either absent or unwilling to attend due to work. When women arrived without a husband, they said they were fined, scolded, or told to wait in a longer queue. In Ntcheu North, women said they needed a letter from the village chief to avoid fines or were sent home. Some women said they *“rented”* husbands for MKW 500 (USD 0.62) to avoid fines.

When you go with your husband, you are given priority.

Mother, Ntcheu West

Women were also scolded or fined up to MWK 4000 by health workers for missing sessions or not following advice, discouraging their return. In Ntcheu North, mothers said (after their 3‐4 h trip to the facility) that they could be sent back by the health worker for being late or forgetting their health passport.

- *Iron tablets ran out and misunderstanding of purpose*.
  While some mothers said they received iron tablets at ANC, some reported the facility ran out and asked them to purchase them. Of those that did receive tablets, one said they didn't take them because they didn't know their purpose, they made them feel sick, or they didn't always have food to eat with the tablet.

#### 3.4.2 “In the Past 6 Months, Each of the Two Little Ones Had Diarrhoea Every Month”

Parents across locations reported diarrhoea as the most common health issue for children, which was most prevalent during the rainy season (December to April).

In the past 6 months, each of the two little ones had diarrhoea every month.

Mother, Ntcheu North

Mothers considered diarrhoea to be normal and treated it with traditional remedies—roots, barks and leaves, before trying over‐the‐counter painkillers. A child would only go to a clinic or hospital if very sick, since the clinic was far away. No mother mentioned giving the child more fluids or Oral Rehydration Salts (ORS) when their child had diarrhoea.

When asked what caused diarrhoea, mothers listed a number of causes including unclean areas for babies, improper waste disposal, poor hygiene practices, feeding undercooked food, children putting dirty things in their mouths, not using soap to wash utensils and dishes, animal droppings in the household, unclean bedding, and not washing nappies. However, mothers said they couldn't address all these issues due to their workload and children being cared for by older siblings.

#### 3.4.3 Poor Hygiene in the Households

Table 4 summarises the six hygiene‐related issues identified during household observations, and disease risk factors for babies and young children. These include: lack of handwashing with water and soap at key times, unsafe drinking water, poor food hygiene, open air defecation, inappropriate disposal of baby faeces and close proximity of pests and livestock.

**Table 4:** Household observations and disease risks.

Issue	Description
Handwashing	Handwashing was mainly to remove visible dirt or before and after eating, but without soap, with everyone rinsing hands in the same bowl. Most families didn't have soap at home. Powdered detergent was available in villages, but buying it wasn't a priority due to limited cash. One family in Balaka East noted only those with city relatives sending money regularly had soap. Families with soap used it sparingly for laundry, dishes, and bathing. Only one household had a tippy tap, which wasn't working. No handwashing was seen before handling babies, preparing food, feeding, or after changing nappies. Children weren't encouraged to wash hands and followed family patterns. Families rarely mentioned concerns about bacteria or illness.
Drinking water	In all locations, the family's drinking water came from boreholes or pumped well water, except in one village where the borehole water was *“so salty even animals don't drink it,”* so they preferred shallow unprotected well water. A health worker had given them chlorine tablets to treat the well water, but they didn't use them. All families felt their water was safe, and no families boiled or treated it before drinking. Young children and babies as young as 2 months were given the same water in the same cups as the rest of the family.
Food hygiene	Meals were eaten on kitchen mud floors or outside, often near animals. Vegetables were usually unwashed before cooking, as families said washing made them watery. Dishes were washed directly in the stream or in basins next to the house with stream or rainwater, generally without soap but occasionally with ash. Dirty dishes and cooking utensils sat after a meal and were washed before the next meal. No special measures were taken to wash bowls, plates, or cups used by babies or young children, who used the same utensils. Dishes dried on a wooden rack or on the ground outside or in the grass.
Defecation	Pit latrines were often shared by 3‐5 households. Many low‐quality latrines with leaf roofs were destroyed by Cyclone Ana and hadn't been rebuilt. One family said their latrine collapsed every rainy season, so they rebuilt it during the dry season and used a neighbour's latrine in the meantime. While all households reported using latrines, open defecation was common, especially for children and adults working in fields. In Balaka East and Ntcheu North, children defecated in the bathing area outside the house, and parents threw the faeces into a nearby field or garden. Children under 3 were never seen using latrines in any visited household. Some wore no clothes and defecated as needed, others wore plastic underwear. Children over 3 preferred defecating outside due to the smell or poor condition of latrines. Parents said young children were free to defecate outside the house.
Baby faeces	Mothers in two locations said they didn't consider baby faeces dirty or unhygienic until babies were at least six months old. We observed soiled nappies or clothes washed with other clothes in a nearby stream or a basin near their homes. The basin was often used to wash dishes. In Balaka East, mothers washed soiled nappies in the stream with other children's clothing, without emptying the nappies first. One mother said she didn't worry about others using the water downstream, as everyone does the same. Only a few households used small amounts of soap to wash their clothing or nappies.
Pests and livestock	Many flies settled on dishes and kitchen utensils. Mothers said flies intensified in the dry season due to rubbish, open defecation, and animal droppings nearby, but viewed this as an irritation rather than a hygiene risk. Large rats, ticks, and cockroaches were observed around households. Many families kept goats and chickens inside or near the house to prevent theft. Families occasionally swept animal faeces and tossed them into the garden. None of the visited families were concerned about hygiene risks from animals around the house. In Ntcheu West, one family lived near a pig farm, and said that in the rainy season, pig faeces washed into the river used for bathing and washing dishes.

### 3.5 Responsive Care

#### 3.5.1 “You Are the One Who Is Pregnant, You Want the Baby, so You Have to Face It”

In all households, mothers were the main carers of babies, with grandmothers sometimes helping with babies over 6 months. Mothers said childcare was their responsibility because they chose to have the baby.

You are the one who is pregnant, you want the baby, so you have to face it.

Breastfeeding mothers, People Driven Design, Ntcheu West

Women mentioned that their heavy workload limited their ability to play with their children, so children over 1 year played with older siblings. Babies under 6 months were carried on the mother's back or left on mats. Female family members, siblings, or neighbours were occasionally seen interacting with babies, but this was rare. Except for one or two mothers who were seen singing or praising their child, most mother‐child interaction was limited to giving instructions like *“eat more”* or *“stop crying”*. During feeding, caregivers put the spoon in the baby's mouth without speaking or just said *“eat, eat.”* Children under 3 years fed themselves from their small plate, while older children used their hands to pick from the larger family plate. Both parents were observed using physical punishment on children as young as three, and a 4‐year‐old was even seen being hit with a stick.

#### 3.5.2 Fathers Are Absent or Disengaged

Fathers were often absent due to work and only helped with childcare when mothers were busy, stressed, or they were *“forced.”* Out of 12 households, only one father was seen playing with his baby over the 4‐day immersion period. Fathers were generally available in the mornings and evenings but said they preferred socialising with other men or felt awkward holding babies and unsure how to care for them.

### 3.6 Learning Opportunities

#### 3.6.1 Children Play With Peers and Homemade Toys

In the study households, researchers observed that toys were rare and were made by children themselves from mud, grass or other materials available. Children played mostly alone or with peers, with little adult interaction or supervision, since mothers were busy with chores, and fathers were absent or disengaged. Children of all ages (siblings and from neighbouring houses) played together outside the house or in nearby fields, and toddlers old enough to stand joined in or watched older children.

CBCCs – community‐based preschools ‐ existed in all locations, but only one was active during the study. CBCCs closed when communities were no longer able to provide porridge to children or pay CBCC volunteer allowances. Mothers valued the CBCCs mainly for the food provided, as children would not have breakfast otherwise ‐ “*kids just go there to eat.”* The CBCCs visited had no latrines, no decorations, toys or outdoor play equipment, but mothers said children did learn the alphabet and calendar and primary teachers said that children who attended CBCCs were *“more active and learned new things more easily.”*

### 3.7 Safety and Security

#### 3.7.1 Adolescent Pregnancy Is Common and Seen as Normal

The main safety issue observed, was related to adolescent girls. Researchers across the four locations, observed many adolescent girls who were either pregnant or had young children. People said that adolescent pregnancy was normal, and that many girls would have their first child at 15–16 years and become grandmothers in their 30s. Girls that researchers met, said they left school after primary school due to high schooling costs and because they did not see education as relevant for their future as farmers. Once out of school, marriage and children were viewed as respected ways to occupy time and be financially supported (by the husband), especially when parents struggled to support them.

#### 3.7.2 Young Mothers Are Particularly Vulnerable

Young mothers faced more challenges than their peers or older mothers, because they were less likely to have their own business and were less able to work outside the village due to caregiving responsibilities, which made them more financially dependent on their husband and families for support. Husbands often worked outside the village in better‐paying jobs, leaving young mothers with their families. One mother in Balaka East even said that “*girls did not have enough to eat.”* Adolescent pregnancy wasn't seen as risky, but stories of young girls having miscarriages or needing caesareans were attributed to them *“being small.”*

## 4 Discussion

This study found that the five nurturing care domains essential for children to survive and thrive are not being met in the four locations studied. The main issues identified include poor maternal, child, and family diets (two meals per day of nsima and boiled leaves); inadequate infant and young child feeding practices (early introduction of fluids and food, inadequate complementary feeding); poor hygiene environment and practices (lack of handwashing with soap, poor management of faeces, lack of food hygiene, close proximity to animals, and inadequate diarrhoea management); low use of health and nutrition services (ANC, PNC, GMP); limited responsive care, child stimulation, and learning opportunities; and early pregnancies. These are influenced by wider and more systemic issues including chronic poverty, food insecurity, economic and climate shocks (erratic weather and poor soil fertility which affect crop production and households' ability to purchase nutritious foods and access services); poor access and quality of health and nutrition services (too far away, discriminatory and not useful), limiting women's ability and willingness to use those services; gender inequality (absent and disengaged fathers, with chores and childcare falling on women) affecting women's ability to breastfeed and adequately care for themselves and their child; and harmful social norms and expectations (combined with poverty) that lead girls into early marriage and pregnancy, putting their and their children's survival and health at risk.

This study is the only study in Malawi that looks across the nurturing care framework and unpacks the complex drivers of caregiving behaviours across the five nurturing care domains. The large number of quantitative studies (summarised in the introduction) that have looked at factors associated with stunting in Malawi support the findings of this study, by identifying maternal and caregiver factors, gender inequality, household food insecurity, child health and disease and inadequate caregiving factors (hygiene, infant and young child feeding and low use of services) as potential risk factors for stunting. A contemporaneous study conducted in the same locations found that caregiver diet, empowerment, and mental health were significantly associated with child development outcomes (Bliznashka et al. 2024). The more limited number of qualitative studies also support the findings of this study, identifying poverty, food insecurity, maternal wellbeing and gender inequality as key determinants of caregiving practices (Gladstone et al. 2018; Mseu et al. 2014; Mkandawire et al. 2022). This study goes deeper to explain how these factors influence caregiving practices and child growth and development in Malawi and reinforces the recommendation for a contextual multi‐sector approach to prevent malnutrition and improve child development.

The study had some limitations. The first is linked to the field researchers, who were NGO or district government technical staff, rather than experienced RCA researchers, with little prior experience of qualitative research methods, and in‐depth data gathering methods which may have limited the depth of information. This was a concern particularly for the more sensitive topics like household gender dynamics and gender‐based violence, which was not identified in any locations, because these researchers were less able to probe sensitive issues. The main reason for having NGO and government staff as researchers was so that they would gain a deeper “lived” experience of the issues faced by the households they serve. To minimise these limitations, the researchers had a week of practical training (role modelling conversational and participatory research techniques) led by a team of Empatika RCA researchers, who then mentored them during the immersion research, and facilitated the 2‐day debrief sessions to capture all relevant information.

Another limitation may be social desirability bias, as households adapt their behaviours to the researchers living with them. This risk may have been amplified by the researchers' affiliations with NGOs and government institutions, which could reinforce existing power dynamics. To minimise this risk, the researchers were trained to interact with households in a way that minimises power distances. They explained that their visit aimed to understand and experience households' daily life, emphasised the need to carry on their lives as normal and explained that all conversations and observations would be shared anonymously. Spending 4 days and nights with the households also helped reduce social desirability bias and power imbalances ‐ including gender related ones with male researchers‐ by fostering more informal relationships with household members, and making it difficult for households to adapt their behaviours for that length of time.

Another limitation was the absence of men in the households visited, as many worked outside the villages. Most interactions were with women, children, and the elderly, reflecting their perspectives. Researchers also struggled to get men to join the People Driven Design workshops. Men's perspectives are therefore largely missing from the study findings.

There are also some RCA‐related methodological limitations, particularly around data rigour and accuracy, since conversations and observations are noted later and in private, not recorded or transcribed on the spot. This approach leaned on researchers' memory and their ability to share discussions accurately with the Empatika team during the debrief sessions. To address this, researchers noted observations and conversations in their notebook whenever possible (often in the evenings) and used the Areas of Conversations memory aid to track topics discussed. Debrief sessions happened right after the research, with researchers grouped by location to aid recall and triangulation of findings. The RCA method also focuses on households and the people and services household members interact with, possibly overlooking broader community issues and power dynamics. However, by spending extended time in the community and working in small teams, RCA can also capture wider community issues and provide unique insights from people's perspectives, often missed by traditional research methods. These unique insights into people's daily lives are valuable for understanding complex behavioural drivers, which this study was largely able to do in relation to the five nurturing care domains.

A final limitation is the restricted geographic and temporal scope of the research: data were gathered in only two districts of Malawi, and solely during the lean season, which limit the generalisability of the findings to other parts of Malawi and to other seasons. Conducting similar research in additional districts and at different times of the year would yield a more comprehensive, representative picture of the factors that shape caregiving behaviours across the country.

## 5 Conclusion

This study found that chronic poverty and food insecurity, economic and climate shocks, limited access to quality health, nutrition and ECD services, poor maternal wellbeing, gender inequality and harmful social norms are key determinants of nurturing care behaviours in the four locations studied. This study underscores the need for a multi‐sector approach to improve maternal and child nutrition and development. It also stresses the importance of understanding the contextual determinants of malnutrition to inform multi‐sector programmes and policies and meet Malawi's SDG and WHA targets for maternal and child health, nutrition, and development. Unless these determinants are addressed, caregiving practices are unlikely to improve, and Malawi will not progress on meeting its nutrition and child development objectives (National Economic Council 2000; Government of Malawi 2017; Government of Malawi et al. 2018) and will fail to meet its SDG and WHA targets on nutrition and maternal and child health and development (SDG 2, 3, 4).

## Author Contributions

N.R., D.J., D.S., B.P., J.N., P.L., A.G., M.G. designed the research. N.M., P.K., R.G., S.M., S.S., F.Z., C.L., J.M., B.K., A.K., S.C. performed the immersion research. N.K., D.T., D.H., I.A., R.A., Y.I., S.E. led the training and supported the analysis of the research. O.K., M.G. provided technical support. D.S. and D.J. analysed the data. N.R., D.S. and D.J. wrote the paper. All authors read and approved the final manuscript.

## Conflicts of Interest

The authors certify that they have no affiliations with or involvement in any organisation or entity with any financial interest (such as s honoraria; educational grants; participation in speakers' bureaus; membership, employment, consultancies, stock ownership, or other equity interest; and expert testimony or patent‐licensing arrangements), or non‐financial interest (such as personal or professional relationships, affiliations, knowledge or beliefs) in the subject matter or materials discussed in this manuscript.

## Supporting information

Supplementary Material.

## Data Availability

The data that support the findings of this study are available on request from the corresponding author. The data are not publicly available due to privacy or ethical restrictions.

## Endnotes

1 Each household was given 5 kg respectively of rice, sugar, beans, groundnuts and corn soya blend, salt and 10 tablets of soap (5 for bathing, 5 for clothes washing).

2 Exchange rate on 1/3/22.
