# Supplementary material for: Determinants of Nurturing Care Behaviours in Malawi: An Ethnographic Study
Source: Matern Child Nutr. 2025 Jul 27;21(4):e70076. doi: 10.1111/mcn.70076 (PMC12454197; doi:10.1111/mcn.70076)
Supplement: Supplementary file 1 — Supplementary Material. [file MCN-21-e70076-s001.docx]

**Supplementary material 1: Researcher characteristics**

|  | **Sex** | **Age** | **Highest education** | **District come from** | **Affiliation at time of study** | **Affiliation now** |
| --- | --- | --- | --- | --- | --- | --- |
| 1 | F | 38 | BA Social Science in Dev Studies | Blantyre | MEAL Officer, Save the Children, Lilongwe, Malawi | MEL Country Lead, PATH |
| 2 | F | 32 | Bachelors of Tourism Management | Ntcheu | Dept of Trade, Ntcheu District Council | Farmer |
| 3 | M | 27 | Bachelor of Science food science and technology | Ntcheu | Agriculture | DAEC |
| 4 | F | 32 | Bachelor’s degree in social work | Nkhotakota | Save the Children | Save the Children |
| 5 | F | 30 | BA Human Sciences and Community Services, LUANAR | Neno district | Nutrition Officer, Ntcheu District Council | Field Officer, Give Directly |
| 6 | M | 38 | BAEd Humanities, MA Health Behaviour Change Communications (Pending) | Zomba | SBC Technical Coordinator  Save the Children | SBC Manager, Save the Children, Titukulane |
| 7 | M | 40 | Dip Food Nutrition and Livelihood Security | Dowa | District Project Facilitator, Save the Children, Balaka | Project Facilitator, Save the Children, Balaka |
| 8 | F | 35 | Advanced Diploma in Community Development | Blantyre | Balaka District Council-Social Welfare office-Intern | NA |
| 9 | M | 25 | Degree in Business administration (3ry student) | Mchinji | Intern- Information Communications, Technology Department-Balaka | NA |
| 10 | M | 38 | BA of Social Science | Ntcheu | MEAL Coordinator, Save the Children, Balaka | MEAL Coordinator, Save the Children, Balaka |
| 11 | F | 43 | BSc, Social work and Community Development | Kasungu | Project Gender Facilitator, Save the Children, Ntcheu | Project Gender Facilitator, Save the Children, Ntcheu |
| 12 | M | 27 | Diploma in Community Development | Kasungu | Balaka Community Development Office | Not working |

**Supplementary material 2: Households that didn’t want to take part**

| **Researcher** | **# not willing to participate** | **Reasons households (HH) were not willing to host a researcher** |
| --- | --- | --- |
| 1 | 1 | Most HH members were men so were not comfortable accommodating a woman |
| 2 | 2 | HH members were scared of hosting a stranger  HH members suspected the researcher to be a satanist. |
| 3 | 3 | Woman headed HH, not comfortable man when husband lived abroad.  HH suspected him of being a thief  HH suspected of being blood sucker. |
| 4 | 1 | House too small to accommodate researcher, not enough food to feed the researcher |
| 5 | 0 | The first HH agreed to participate |
| 6 | 0 | The first HH agreed to participate |
| 7 | 1 | House was too small |
| 8 | 3 | Husband was not around and mother could make a decision on her own.  HH members afraid to host a stranger, and were not warned  HH did not have enough food to feed the visitor, and they cannot give food to someone from the city. |
| 9 | 3 | HH belonged to the chief who felt that hosting the researcher could raise suspicions among other villagers  HH did not have enough food to host the researcher  Wife was working away so not able to host |
| 10 | 2 | HH not ready to host due to hunger |
| 11 | 2 | HH not comfortable to host a visitor because unsure of intentions in the community  Not enough space to keep a visitor |
| 12 | 3 | HH members suspected the researcher of being ablood sucker  HH did not have enough food to host the researcher  House was too small to host the researcher |

**Pregnant women care and nutrition**

Perceptions of own health and nutrition status**; local norms** re good health & wellbeing for pregnant women (healthy weight gain, signs of good health, physical exertion (workload)/rest). Diet & changes made for pregnancy (observations of dietary diversity/quantities eaten) Views of, access to and use of **antenatal care** (nature of) reasons to miss ANC; perception of advice provided/comms materials used at ANC/home visits; receipt and understanding of supplements, fortified food, immunization, malaria prevention advice/commodities. Own purchase of supplements. ‘Good’ and ‘bad’ foods for pregnant women. Understanding of **risk factors** (e.g. anaemia). Adolescent pregnancy – understanding of care, risks etc. Religious**/spiritual/old time beliefs** and persisting traditions related to pregnant women. Perceptions of what is a good birth weight. Fathers views on care in pregnancy and their role in providing additional support

**Quality/appropriateness of maternal/child health services and support groups** Different types of support available; Knowledge/attitudes and practice of informal & formal service providers towards mothers (trust, friendliness, time, patience etc); home visits; nature of engagement; other sources of information (TV, radio, older women, other influencers (Church, school) etc) -trust/authority. Disconnects between information/advice provided & practice; nature of interactions (Counselling vs telling/reprimanding etc) interaction with adolescent/single mothers, **nature and value of support provided during 1000 days**/frequency of interactions; mothers/fathers perceptions of health providers and HDWs. Motivation/incentives for health staff to support ‘first 1000 days’.

**Supplementary material 3: Areas of conversation**

**Food- diversity /preparation**

Availability/diversity/affordability/accessibility (seasonal variation); food preparation & storage; Family eating (frequency, content, dynamics); views on life cycle related dietary needs; food preferences; changing trends (e.g use of convenience foods) use of VSL for food purchase/cultivation; availability of fortified seed varieties etc

**WASH environment for babies and infants**.

Household and community sanitation practice, norms, preferences. Water sources, special preparation and storage for babies; Perceptions on cleanliness (hygiene vs dirt vs ‘smelling nice’), perceptions of what hygiene provisions are needed for baby/infant wellbeing; understanding of /protection from risk factors (pathways for pathogens); hygiene around food preparation for infants. Disposal of baby faeces, washing soiled clothing; Views (positive /negative) of living in proximity of animals.  Teething hygiene, care-givers hygiene (hand washing/nipple washing, toilet practices).. (Triangulate all this with observations)

**Feeding and care babies and infants under 3**

Actual practice of **exclusive breast feeding** in first 6m; views on breastfeeding- frequency/ timescale (nutrition vs. comfort/pacifying), hygiene; views and use of milk formula (if used) hygiene practices around bottle use; **introduction of other foods** (why, what, when and how). Homemade vs purchased food. Diversity of diet/quantities, consistency of food, flavourings, seasonality (cashflow, availability prices etc) affecting diversity of diets. Views on feeding practices (were, frequency, interaction (responsiveness, child-led, gender differences). Views on snacks response to child’s food demands; basis of feeding practices- convenience, comfort, taste, availability/accessibility of food, desirability (individual and social desirability- ‘*perceptions of being a good mum’*). Views on responsive feeding, play, stimulation, being outside (vit D). Perception of good role models, others who struggle. Feeding ‘picky eaters’, sick children, those with disabilities. Role of CBCCs- substitution /supplementary feeding

**Attitudes/motivation for behaviour change**

improvements aspired to (priorities); future dreams and plans for themselves and children, concerns, worries for the future, what does good change look like, what is helping/hindering change, changes over time- best and, worst, coping with changes, ‘what if’ scenarios, consequences. Role models- whom do caregivers aspire to . Support for change needed; likely motivators/influencers
